# Proton-Coupled Electron and Energy Transfer in Molecular Triads

**DOI:** 10.1021/acs.accounts.6c00068

**Published:** 2026-04-17

**Authors:** Sharon Hammes-Schiffer, James M. Mayer, Leif Hammarström

**Affiliations:** † Department of Chemistry, 6740 Princeton University, Princeton, New Jersey 08544, United States; ‡ Department of Chemistry, 5755 Yale University, New Haven, Connecticut 06520-8107, United States; § Department of Chemistry, Ångström Laboratory, 8097 Uppsala University, Box 523, 75120 Uppsala, Sweden

## Abstract

Electrons and protons are the simplest particles in chemistry, and their transfers are among the most fundamental chemical reactions. It is increasingly recognized that these two particles often transfer in the same elementary kinetic step, resulting in the most common type of proton-coupled electron transfer (PCET). PCET has evolved from a curiosity to a major research field that is central to a broad range of processes in chemistry, biology, and materials science.

PCET evolved from electron transfer, in both its experimental and theoretical origins. One wonders how the field would be different if it had been called electron-coupled proton transfer. This equivalent terminology illustrates that the proton is on equal footing to the electron, making PCET perhaps the simplest case where the quantum properties of both an electron and a nucleus need to be considered.

The fundamental understanding of PCET in solution builds on the remarkably impactful theory of electron transfer (ET) developed by R. A. Marcus and others. At a basic level, ET theory is marked by a quadratic dependence of the reaction barrier on the reaction free energy (Δ*G* ^⧧^ on Δ*G°*), with normal and ‘inverted’ regions separated by a barrierless region (Δ*G* ^⧧^ = 0), plus an electronic coupling that determines the electron tunneling probability. The theory for PCET includes additional essential elements: the quantum mechanical treatment of the transferring proton(s) as tunneling particles, multiple channels corresponding to reactant and product electron–proton vibronic states, vibronic coupling rather than electronic coupling, and a distribution of proton donor–acceptor distances.

Our recent studies of ultrafast intramolecular PCET in molecular triads were the first to demonstrate the corresponding free-energy dependence for PCET, including the inverted region. Inverted behavior was previously thought to be difficult to observe experimentally for PCET because it connects vibronic states rather than electronic states. Due to the more closely spaced vibronic state energy levels compared to electronic state energy levels, there is usually a nearly barrierless pair of reactant and product vibronic states that obviates the inverted region. For these molecular triads, however, the vibronic coupling is very small for the barrierless pair, allowing observation of the hallmark inverted region.

While looking for ultrafast PCET, we discovered a new elementary chemical reaction that we denoted proton-coupled energy transfer (PCEnT). In PCEnT, proton transfer (PT) is coupled to electronic excitation energy transfer. As with PCET, PT is required for the reaction to be thermodynamically accessible. In our molecular triads, PT occurs within the phenol–pyridine acceptor unit, concerted with electron transfer to a photoexcited anthracene (PCET) or electronic excitation energy transfer from a photoexcited anthracene (PCEnT). The dominant reaction depends on the molecular substituents and reaction conditions. A theory for PCEnT with some of the same essential elements as PCET theory, along with some fundamental differences, has been developed and applied to a triad system.

## Graphical abstract

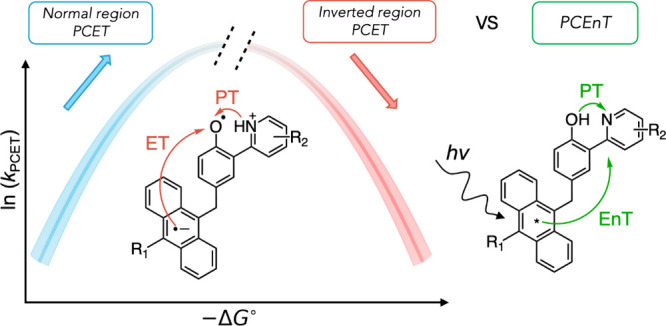

## Key References

-

Parada, G. A. ; Goldsmith, Z. K. ; Kolmar, S. ; Pettersson Rimgard, B. ; Mercado, B. Q. ; Hammarstrom, L. ; Hammes-Schiffer, S. ; Mayer, J. M. Concerted proton-electron transfer reactions in the Marcus inverted region. Science 2019, 364, 471–475 30975771 10.1126/science.aaw4675PMC6681808.^13^ *The first demonstration of the inverted region in the free-energy dependence for proton-coupled electron transfer (PCET)*.
-

Pettersson Rimgard, B. ; Tao, Z. ; Parada, G. A. ; Cotter, L. F. ; Hammes-Schiffer, S. ; Mayer, J. M. ; Hammarstrom, L. Proton-Coupled Energy Transfer in Molecular Triads. Science 2022, 377, 742–747 35862490 10.1126/science.abq5173PMC9597948.^14^ *The discovery of a new mechanism, denoted proton-coupled energy transfer (PCEnT), is reported.*
-

Cui, K. ; Hammes-Schiffer, S. Theoretical Insight into Proton-Coupled Energy Transfer in an Anthracene-Phenol-Pyridine Triad. J. Am. Chem. Soc. 2025, 147, 21708–21717 40493791 10.1021/jacs.5c03866.^42^ *Application of the recently developed theory for PCEnT to one of the triads as the first application of this theory.*

## Introduction

Proton-coupled electron transfer (PCET) reactions underlie a wide range of chemical and biological processes, from catalysis and energy conversion to regulation of circadian rhythms.^1–9^ With a simultaneous transfer of protons and electrons, PCET facilitates charge transfer reactions that would otherwise be energetically unfavorable. At the same time, the coupling of electron transfer (ET) and proton transfer (PT) leads to more complexity compared to their separate reactions. Today, the term PCET includes both concerted and sequential reactions (ET-PT or PT-ET), and it can be challenging to distinguish and experimentally control which path the reaction follows. The stepwise reactions can be described by the separate theories for ET and PT, while distinct theories have been developed for the concerted reaction.^1,5,10–12^ In these theories, the driving-force dependence of the rate constant is of central importance, especially in the context of PCET reactions for efficient energy conversion and the potential for concerted PCET to avoid high-energy intermediates. In our collaborative studies of molecular triads for photoinduced, intramolecular PCET, we wanted to investigate the driving-force dependence over a wide range. As a result, we found the first demonstration of the Marcus inverted region behavior for concerted PCET,^13^ where the reaction rate decreases with increasing driving force, which is important for processes such as efficient charge separation in solar energy conversion. We also found that important properties of the vibronic manifold of states involved in the reaction dictate whether the Marcus-type quadratic free-energy dependence will be observed or not. Moreover, we discovered a new type of elementary chemical reaction, which we denoted proton-coupled energy transfer (PCEnT).^14^ These studies have given us unexpected insights, as summarized in this Account.

## Free Energy Dependence of PCET: Theory

The description of the reaction coordinate and the resulting reaction barrier (Δ*G* ^⧧^) was the central achievement of Marcus in developing ET theory.^15,16^ The parabolic dependence of Δ*G* ^⧧^ on the reaction driving force (−Δ*G°*) leads to the famous behavior where the ET rate constant first increases with increasing driving force until the reaction is barrierless, when the driving force equals the reorganization energy (−Δ*G°* = λ), after which the rate constant decreases again in the so-called inverted region (−Δ*G°* > λ). This behavior is shown for nonadiabatic ET in Figure 1 and in the following equation:

1a $$k_{\mathrm{ET}}\propto V_{\mathrm{ET}}^{2}\;\exp\left[-\Delta G^{⧧}/(k_{B}T)\right]$$

1b $$\Delta G^{⧧}=\frac{{\left(\Delta G^{o}+\lambda \right)}^{2}}{4\lambda }$$

**Figure 1:**
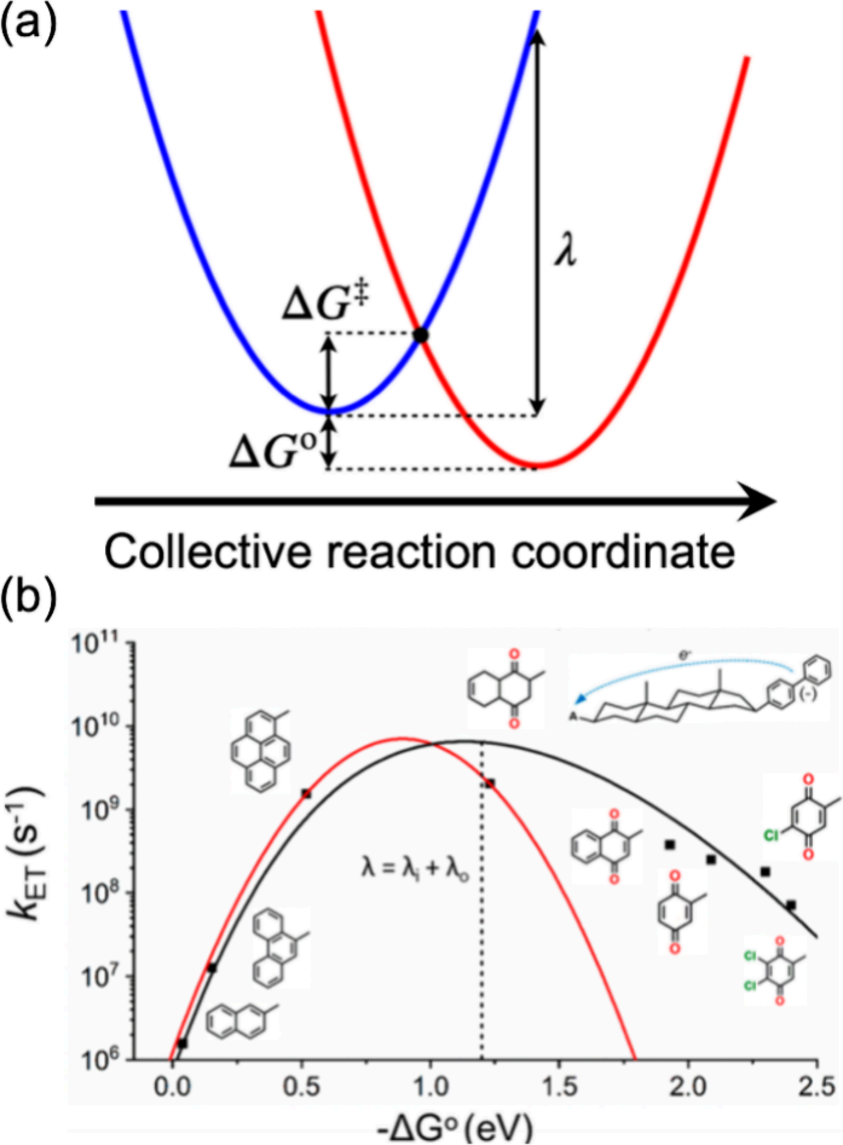
Schematic representations of Marcus ET theory. (a) Parabolas describing the free energy versus a collective reaction coordinate for the reactant state (D + A, blue) and for the product state (D^+^ + A^–^, red). ET from the donor D to the acceptor A leads to the product state with different molecular charges and structures, and the resulting changes in solvent polarization and solute structure are the origin of the Marcus reorganization energy (λ) for ET. (b) The free-energy dependence of *k*_ET_ predicted by Marcus (red curve, from eq 1) and experimentally verified in many studies, but for the first time in ref 20 (black squares). The black curve also includes contributions from vibronic effects, which are typically only important in the inverted region or at very low temperatures. Panel b is reproduced with permission from ref 21. Copyright 2019 American Chemical Society.

Concerted PCET theories contain a related parabolic dependence of Δ*G* ^⧧^ on −Δ*G°*, so a similar free-energy dependence could be predicted for concerted PCET when the other factors remain unchanged.^5,10–12^ This behavior is shown for vibronically nonadiabatic PCET in Figure 2 and in the following equation:^17^

2a $$k_{\mathrm{PCET}}\propto \underset{\mu }{\sum }\underset{\nu }{\sum }P_{\mu }V_{\mu \nu }^{2}\;\exp\left[-\Delta G_{\mu \nu }^{⧧}/(k_{B}T)\right]$$

2b $$\Delta G_{\mu \nu }^{⧧}=\frac{{\left(\Delta G_{\mu \nu }^{{}^{\circ}}+\lambda \right)}^{2}}{4\lambda }$$

However, complications arise from the need to sum over many reaction channels corresponding to different pairs of reactant (μ) and product (ν) vibronic states. The relative contribution of each reaction channel depends on not only the free energy barrier Δ*G* ^⧧^ but also the vibronic coupling *V*_μν_ and the Boltzmann population *P*_μ_ of the reactant vibronic state. The inverted region is observable only for certain combinations of these quantities. Due to the energetic availability of excited product vibronic states, the inverted region may occur only at very large driving forces that are often experimentally unattainable (see Figure 2).

**Figure 2:**
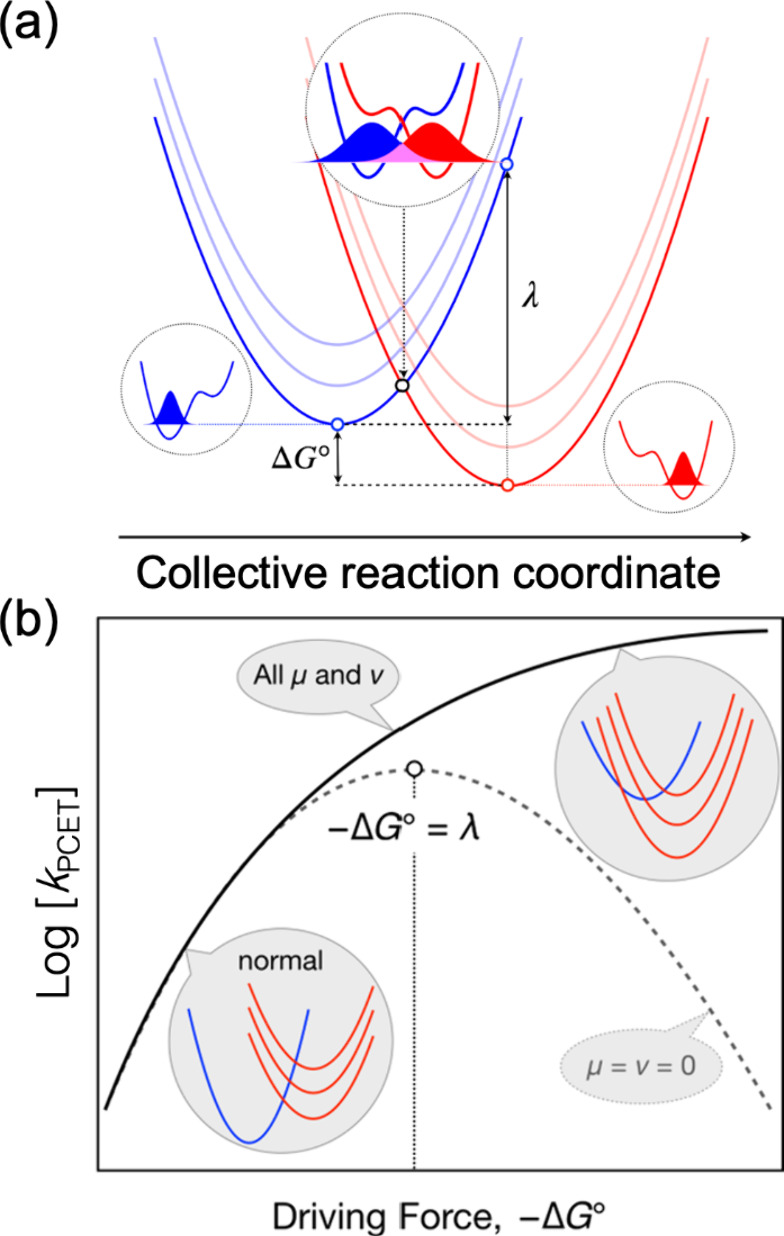
Schematic representation of PCET theory. (a) Parabolas describing the free energy versus a collective reaction coordinate for the reactant (blue) and product (red) vibronic states. Each stacked parabola corresponds to a different proton vibrational state associated with the transferring electron localized on its donor (reactant) or acceptor (product). The proton potentials and the ground state proton vibrational wave functions are shown in black dotted circles. For many PCET reactions, the vibronic coupling for each pair of reactant/product vibronic states is the product of the electronic coupling and the overlap between the proton vibrational wave functions at the crossing point (magenta for the ground vibronic states). Each pair of vibronic states also has a different reaction free energy and therefore a different free energy barrier. The overall rate constant is computed by summing over all pairs of reactant/product vibronic states. (b) The free energy dependence of *k*_PCET_ predicted by PCET theory if only the ground vibronic states are included (dashed black line), exhibiting clear inverted region behavior, or if excited vibronic states are also included (solid black line), which could obviate the inverted region for certain systems. This plot was generated using Morse potentials for the proton potential energy curves, whereas more realistic asymmetric double-well potentials produce qualitatively different results. Panel (a) adapted with permission from ref 22. Copyright 2025 American Chemical Society. Panel (b) adapted with permission from ref 30. Copyright 2019 Royal Society of Chemistry.

Many studies report a normal region behavior, but in almost all cases only a linear dependence of ln­(*k*_PCET_) on driving force is observed. While this is expected when the change in driving force is small compared to 2λ, −Δ*G°* varies by more than 1.0 eV in some of these cases.^18,19^ Therefore, the first experimental evidence for inverted region behavior of concerted PCET^13^ was very important to confirm the driving force dependence for PCET—as it had been 30 years ago as a confirmation of Marcus Theory for ET.^20^

## Design and Photochemistry of Triads

The design of the anthracene-phenol-pyridine triads in Figure 3 (An-PhOH-py) developed out of prior work that showed the phenol-pyridine motif to be a versatile platform for PCET studies. In particular, the strong OH···N hydrogen bond facilitated concerted PCET.^23,24^ With the desire to examine faster, intramolecular PCET, this motif was attached to ruthenium^25^ and then anthracene photo-oxidants.^26^ Dr. Giovanny Parada synthesized a series of compounds with substituents, while maintaining the covalent methylene linkage for good electronic coupling. The key decision to try cyano-anthracene as the photo-oxidant was made initially to enable infrared as well as optical transient absorption studies to identify the PCET products; the higher oxidizing power of the NC-An* excited state was considered a bonus. All these factors were critical for the discovery of the PCET inverted region.

**Figure 3:**
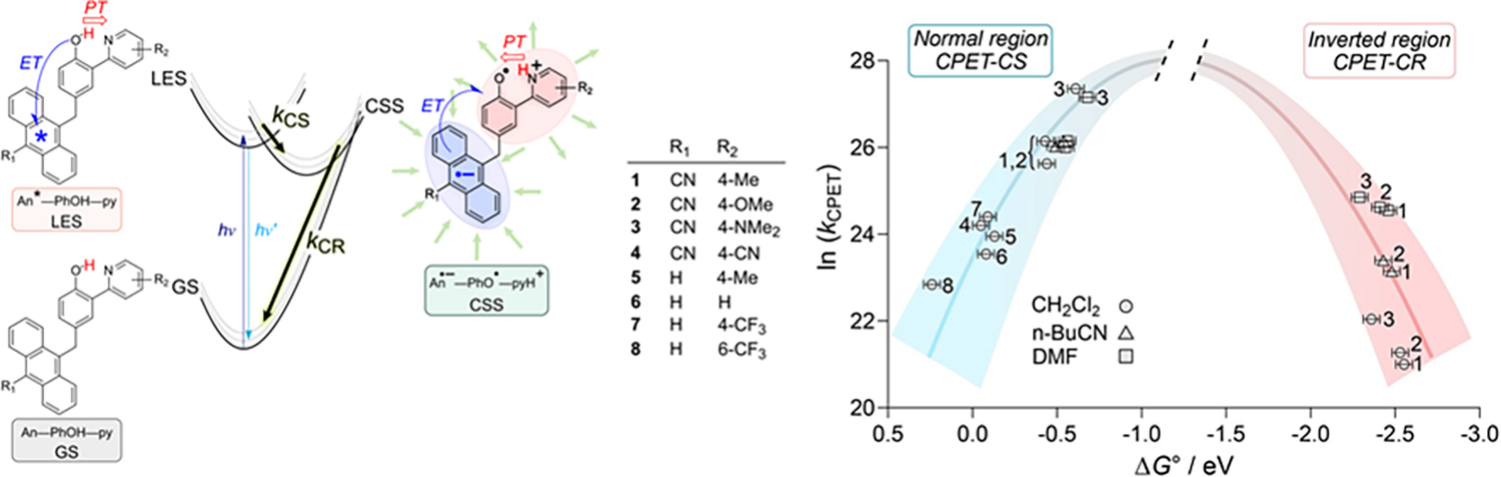
Structures of the triads and schematic of the PCET reactions with kinetic data. Electron transfer to the anthracene−localized excited state (LES) from the phenol, concerted with proton transfer to the pyridine, leads to the charge separated state (CSS), with moderate driving force. The subsequent charge recombination to reform the ground state (GS) is slower, and the large driving force puts it in the Marcus inverted region. The plot of the resulting rate constants is shown, with data in dimethylformamide (squares), butyronitrile (triangles) and dichloromethane (circles). Adapted from ref 13. Copyright the American Association for the Advancement of Science, 2019.

The chemical processes after light absorption were followed by femtosecond transient absorption (TA) spectroscopy in both the visible and mid-IR regions. After initial formation of an An*-localized excited state (LES), triads **4**−**8** rapidly converted back to the ground state (GS) without evidence of another intermediate. For triads **1**−**3**, however, NC-An* decayed to a new spectrum that indicated formation of both the 9-cyanoanthracene radical anion (NC-An^•–^) and a phenoxyl radical. These spectra thus implied NC-An* reacting by PCET charge separation (CS) to form the charge separated state (CSS), by ET from the phenol to An* concurrent with PT from the phenol to the pyridine base (Figure 3). In polar solvents, the CSS decayed without forming additional species, indicating clean charge recombination (CR) with return of the *e* ^–^ and H^+^ from the An^•–^ and pyH^+^ to the phenoxyl radical. Thus, both CS and CR were overall PCET.

Both CS and CR were determined to be concerted PCET rather than stepwise PT-ET or ET-PT using various experiments and analyses. For example, the variation in the rates of decay of the CSSs for **1**−**3**, which have different pyridine substituents, was informative. Rate-limiting ET from NC-An^•–^ to PhO^•^ would have been expected to be relatively insensitive to the pyridinium (X-pyH^+^) substituent, and initial PT from X-pyH^+^ to PhO^•^ was ruled out by the observation that the rate constant increased with *decreasing* acidity of the pyridinium unit for triads **1–3**. For CS, DFT calculations and thermochemical arguments indicated that initial PT or ET are significantly uphill, as had been concluded in the earlier bimolecular thermal studies.^27^ The phenoxyl radical is accessible only if PT and ET occur concurrently. This need to access the easier PhO^•^/PhOH redox couple is the origin of the preference for the concerted PCET mechanism over the perhaps simpler ET- or PT-first pathways.

## Inverted Region Theory Analysis

The theory of vibronically nonadiabatic PCET entails, beyond the Marcus ET treatment, a summation over reactant and product vibronic states (the blue and red stacked parabolas in Figure 2a) to compute the rate constant. The contribution from each pair of reactant/product vibronic states (μ,ν) depends on the balance among three quantities: (1) the Boltzmann population of the reactant vibronic state, *P*_μ_; (2) the vibronic coupling *V*_μv_, which can often be expressed as the product of the electronic coupling and the overlap integral between the reactant and product proton vibrational wave functions (*V*_μv_ = *V* ^el^ *S*_μv_); (3) the free energy barrier Δ*G*_μv_^⧧^ = (Δ*G*_μv_^o^ + λ)^2^/(4λ), where Δ*G*_μv_^o^ is the reaction free energy for this pair of vibronic states and λ is the reorganization energy.

For some PCET systems, the inverted region may not be observed experimentally because of the accessibility of excited vibronic states.^28^ As shown in Figure 2b, if the product is represented by a set of stacked red parabolas, as the driving force increases, the stack of red parabolas is shifted downward. When the crossing point between the blue reactant parabola and a low-lying red product parabola is in the inverted region, crossing with a higher red parabola typically becomes nearly barrierless (i.e., with a free energy barrier of zero). In these cases, a plateau in the rate constant would be observed (solid black line in Figure 2b). Eventually the rate constant will decrease as the driving force increases, but this turnover may not occur in the experimentally accessible regime.

For the triads, our analysis of the contributions to the PCET rate constant provided an explanation for the experimentally observed inverted region behavior for CR. For triad **1**, we found that the (0,3) pair of reactant/product vibronic states dominates the rate constant in dichloromethane (DCM). This crossing point is in the inverted region but contributes significantly because of appreciable overlap between the reactant and product proton vibrational wave functions (Figure 4). In contrast, the (0,7) pair of vibronic states is nearly barrierless, but the overlap between the proton vibrational wave functions is negligible because the seventh excited product proton vibrational wave function is highly oscillatory, leading to phase cancellation with the ground reactant proton vibrational wave function. As experimental support for this picture, the solvent and temperature dependence (298 – 140 K) of the CR rate constant were measured and could be simulated by a model with a single effective barrier with the (0,2) transition dominating in butyronitrile.^29^ Correcting for the energy differences for the dipolar CSS in the two solvents, this is consistent with the (0,3) transition dominating in the less polar DCM.

**Figure 4:**
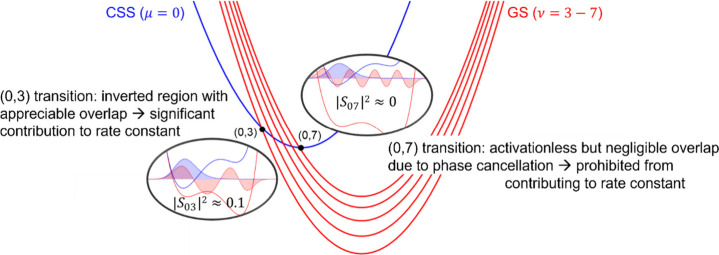
Illustration of the lowest CSS (blue) and multiple product GS (red) vibronic state free energy curves as a function of the collective reaction coordinate for CR in triad **1**. Nonadiabatic transitions associated with a PCET reaction channel can occur at the intersection points between the reactant (μ) and product (ν) parabolas (black dots). Circled diagrams show proton potential energy curves for the reactant (blue) and product (red) and the corresponding proton vibrational wave functions for the dominant reactant/product vibronic state pair (0,3), which has appreciable overlap integral *S*_03_ and contributes significantly to the overall PCET rate constant, and the nearly barrierless vibronic state pair (0,7), which has near-zero overlap integral *S*_07_ and thus does not contribute to the overall PCET rate constant. Adapted from ref 13. Copyright the American Association for the Advancement of Science, 2019.

A more comprehensive computational study of model systems identified two main conditions that should be satisfied to observe inverted region behavior for PCET.^30^ The first condition is that the vibronic state pairs with significant overlap must be in the inverted region, where −Δ*G*_μν_^o^ > λ. The second condition is that the vibronic state pairs that are nearly barrierless must feature prohibitively small overlap. These conditions tend to hold for asymmetric double-well proton potential energy curves with relatively low barriers, such that the midlevel excited vibrational states are above the barrier. This behavior is unlikely to be observed for proton potential energy curves that are single well with a small shoulder or asymmetric double-well potentials with one well much higher in energy than the other well. However, even in these cases, the overlap integrals will eventually become small enough to lead to a turnover in the rate constant for high enough excited proton vibrational states.

It is interesting to contrast inverted region behavior for PCET with that for ET.^28^ For PCET reactions, the proton vibrational motion typically corresponds to relatively high frequencies (∼2000 – 3500 cm^–1^). Since the proton transfers from its donor to its acceptor, the minima of the proton potential energy curves for the reactant and product states are shifted significantly (∼0.5 – 1 Å). In contrast, the vibrational modes coupled to ET are typically lower frequency and are more likely to be approximately harmonic rather than asymmetric double-well potentials. Most importantly, the shift in the minima of these modes for the reactant and product is typically much smaller, ∼0.1 Å. As a result, typically the excited vibrational states of the modes coupled to ET contribute less to the rate constant, which makes inverted region behavior more commonly observed for ET. Still, the inclusion of vibronic effects flattens the parabolic shape in the ET inverted region, as is evident in Figure 1b.^20,31^ An analogous effect is seen in the energy gap law for nonradiative decay of electronically excited states, where a larger excited–ground state geometric distortion gives a weaker dependence on the excited state energy (driving force) energy gap. These issues were studied extensively in the context of ET by Jortner and others.^31,32^

## Inverted Region Demonstration for PCET

The various substituents in the different triads examined (Figure 3) modulated the free energies of the PCET reactions by more than 0.7 eV. For triads **1**−**3**, the pyridine p*K*_a_ values in CH_2_Cl_2_ solvent vary by 2.8 units, a change in Δ*G°* of 0.17 eV. The driving force for CS was therefore 0.17 eV less favorable for **1** (Me-py) than for **3** (Me_2_N-py), and CS was consequentially more rapid for **3**. The CR was instead more favorable for **1**, yet the CR was three times slower for **1** than for **3**. The order of *k*_CR_, **3** > **2** > **1**, is *inverted* from the order of their driving forces (−Δ*G°*_CR_).

The solvent polarity also has a large effect on the energies of the CS and CR processes. While excitation to the LES involves essentially no change in dipole moment of the triad, and therefore is not solvent sensitive, the CSS is stabilized in more polar solvents. This means that more polar solvents make the CS reaction *more* thermodynamically favorable, but the CR starting from the CSS becomes *less* thermodynamically favorable. More polar solvents also lead to higher solvent reorganization energies both for forming and consuming the CSS. For CS, these two effects oppose each other, so the *k*_CS_ is not very solvent dependent, as shown in Figure 3. In contrast, more polar solvents make Δ*G*_CR_^°^ less favorable and λ_CR_ larger, both of which in the normal region would make *k*_CR_ smaller. However, CR is substantially *faster* in the more polar DMF (*ε* = 37) vs CH_2_Cl_2_ (*ε* = 9) by factors of 35, 29, and 17 for triads **1**, **2**, and **3**, respectively. For triad **2** at room temperature, the CSS lifetime (*k*_CR_^–1^) increases from 27 ps in acetonitrile to 2.5 ns in toluene, almost a factor of 100.^29^ The order of rate constants with solvent polarity also demonstrates that the CR reactions are in the inverted region and shows that this can be a large effect. The 2.5 ns lifetime is long enough that bimolecular reactivity from the excited state is possible.

## Proton-Coupled Energy Transfer: Theoretical Prediction

The experiments showing inverted region behavior led to another question: Why is the long-lived transient CSS intermediate and associated inverted region behavior only observed for triads **1**–**3** and not for triads **4**–**8**? The answer was provided by computational methods, using time-dependent density functional theory (TDDFT) to propagate nonequilibrium excited state molecular dynamics trajectories on the S_1_ state following photoexcitation from the equilibrated GS to the anthracene LES.^33^ For triads **1** and **3**, the S_1_ state changed character from the LES to the CSS, with the phenol transferring an electron to An* and a proton to py in a concerted PCET process (Figure 3). In contrast, the S_1_ states of triads **4** and **6** changed character from the LES to another type of state that we called the local electron–proton transfer (LEPT) state—the excited keto-tautomer of the phenol-pyridine, An-[PhO-pyH]* (Figure 5). This state arises from proton transfer from the phenol to the pyridine, along with electron rearrangement within the phenol-pyridine complex, without ET to the anthracene. The anthracene is involved only in the transfer of its electronic excitation energy. The change from LES to LEPT character in **4** and **6** was also observed from potential energy curves generated with the complete active space self-consistent-field method and multireference perturbation theory, including solvent effects with a dielectric continuum model.^34^ The difference can be rationalized by the lower CSS energy in **1–3** compared to **4–8**, due to the cyano group on the anthracene. Since related LEPT states decay rapidly to the GS,^35^ the discovery of this state and the alternative pathway provided an explanation as to why the long-lived transient CSS and inverted region behavior were not detected for some of the triads.

**Figure 5:**
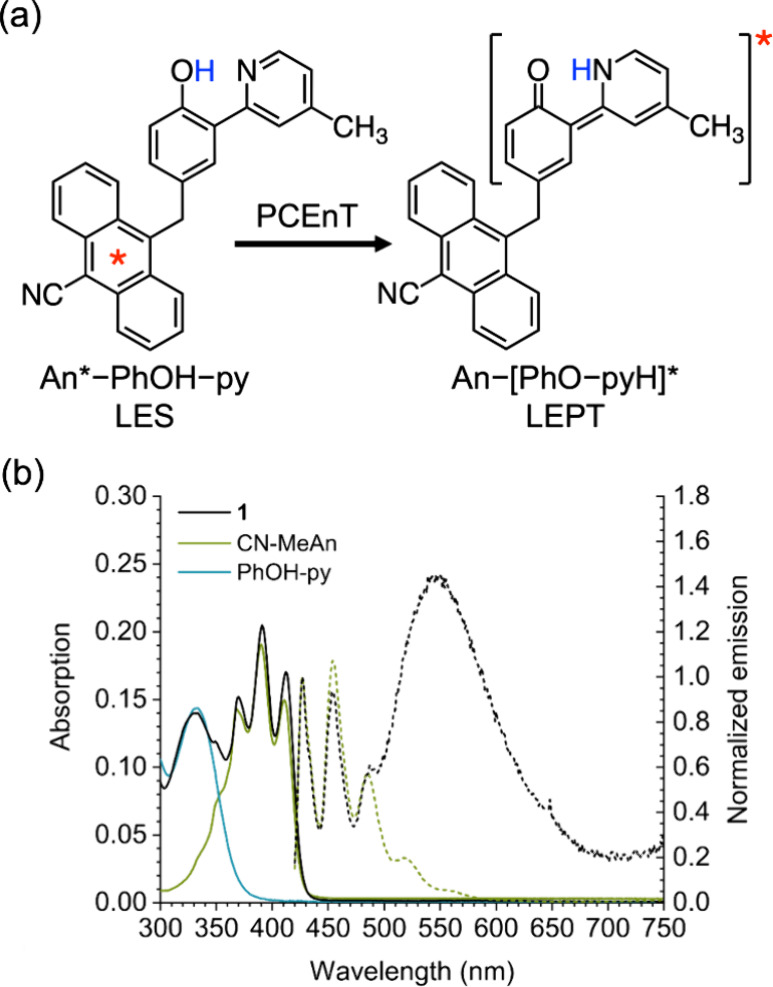
(a) Transition from the LES, with electronic excitation on the anthracene and the more stable enol form of the phenol–pyridine fragment, denoted An*–PhOH–py, to the LEPT state, which is in the excited keto form, denoted An–[PhO–pyH]*, for triad **1**. (b) Solid lines show the room-temperature absorption spectra of triad **1** (black) and its reference components (9-CN-10-Me-An in green, and PhOH-py in blue); the dashed lines show the 77 K fluorescence spectra of the triad (black) and the reference cyanoanthracene (green). Panel (b) adapted from ref 14 under the CC BY license.

These calculations implicated an alternative pathway from the LES to the LEPT state. The LEPT state of the separate phenol-pyridine molecule has long been known to form by direct UV-excitation to *PhOH-py, followed by excited state intramolecular proton transfer (ESIPT in Figure 6).^35,36^ However, the excitation energy used to form the An* was insufficient to populate the *PhOH-py state. The alternative pathway thus involves energy transfer (EnT) from the An* to the PhOH-py unit, coupled to internal proton transfer in the latter.

**Figure 6:**
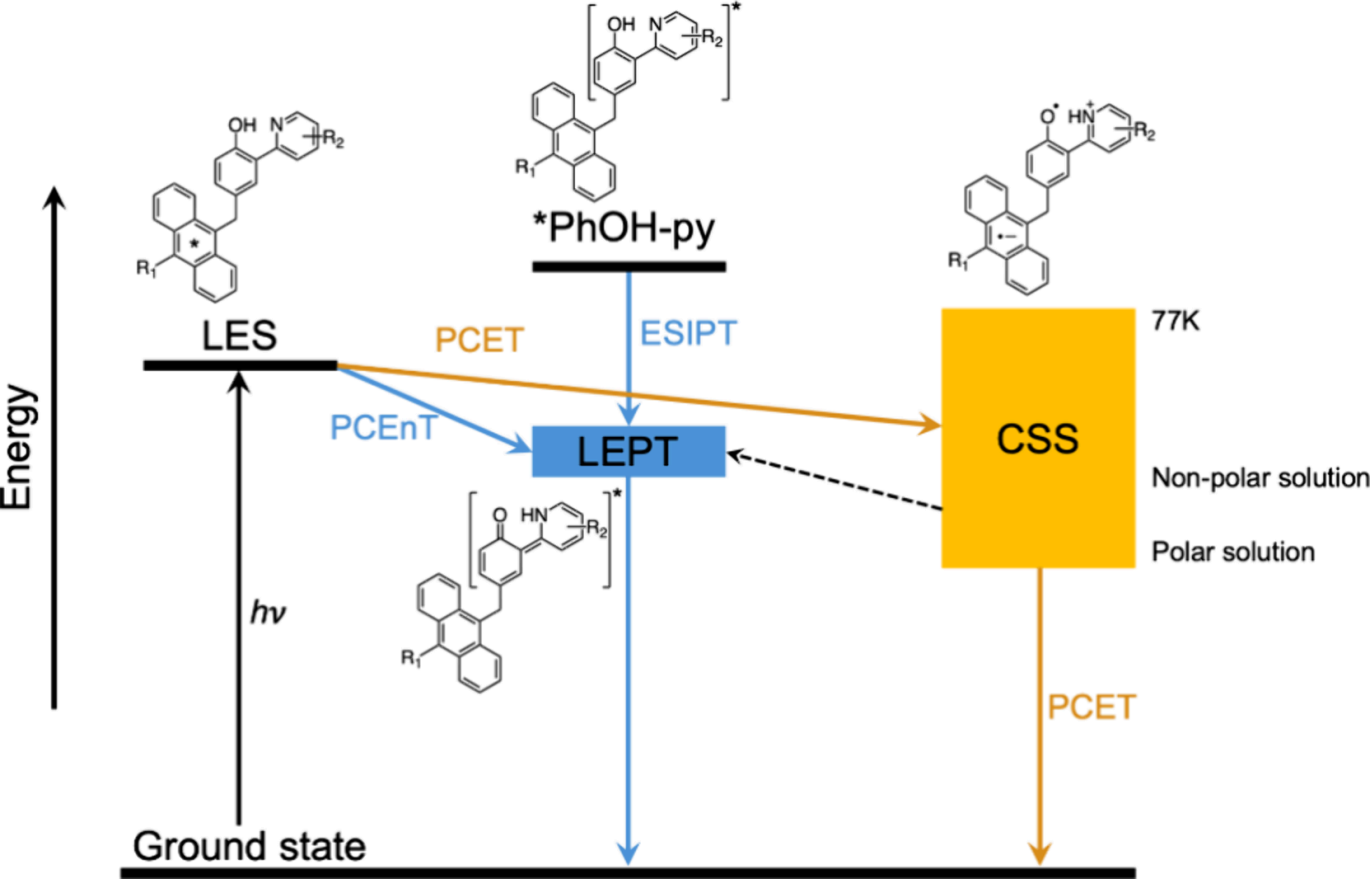
Energy diagram showing the triad states and reactions observed or implicated under different conditions. The CSS energy increases with decreasing solvent polarity and when going to rigid environments (yellow rectangle). The LEPT state with its smaller dipole moment is less sensitive to the solvent environment.

## Proton-Coupled Energy Transfer: Experimental Confirmation

Experimental confirmation of the computations came from further spectroscopic studies of the triads, both in a butyronitrile glass at 77K and in a room-temperature polymer matrix.^14^ Under these conditions, fluorescence from the LEPT state was detected when only the anthracene unit was excited, without any direct excitation of the PhOH-py motif (black dashed curve in Figure 5). Moreover, ultrafast transient fluorescence spectroscopy indicated that the system evolved directly from the LES to the LEPT state, as observed in the calculations. The rigid media were required primarily to make the LEPT state fluorescent and observable, given that in fluid solution twisting of the excited phenol-pyridine unit rapidly quenches the LEPT state.^36^ These rigid conditions also destabilized the CSS relative to the less-polar LEPT state by hindering solvent dipolar orientation and twisting of the An^•–^ relative to the PhO^•^-Hpy^+^, thus diminishing competition from the PCET pathway (Figure 6). Indeed, a reaction to form the LEPT state via the CSS could be excluded because **6** showed a faster PCEnT than **1** (τ ≈44 ps vs ≈69 ps), whereas CSS formation from the LES would have been ca. 0.5 eV less thermodynamically favorable in **6** than **1**.

## Proton-Coupled Energy Transfer as a New Photoprocess

The calculations and experiments thus demonstrate a singlet–singlet energy transfer process, without measurable spectral overlap between the donor (An*) fluorescence and acceptor (PhOH-py) absorption, enabled by the concerted transfer of a proton at the acceptor. Direct energy transfer from the donor (An*) to the enol form of PhOH-py was excluded on energetic grounds, and initial PT in the phenol-pyridine ground state followed by EnT was estimated to be endergonic by ≥ 0.5 eV, thereby incompatible with the ∼ 50 ps reaction time.

This electronic excitation energy transfer in concert with proton transfer is a new elementary reaction, which we denoted proton-coupled energy transfer, PCEnT. Importantly, PCEnT does not involve any charge transfer to anthracene, which remains neutral during this process (Figure 7). The term PCEnT was used previously to describe EnT that is modulated by a hydrogen bond or a protonation state of the reactant, but this is a fundamentally distinct mechanism because it does not entail proton transfer.^37,38^

**Figure 7:**
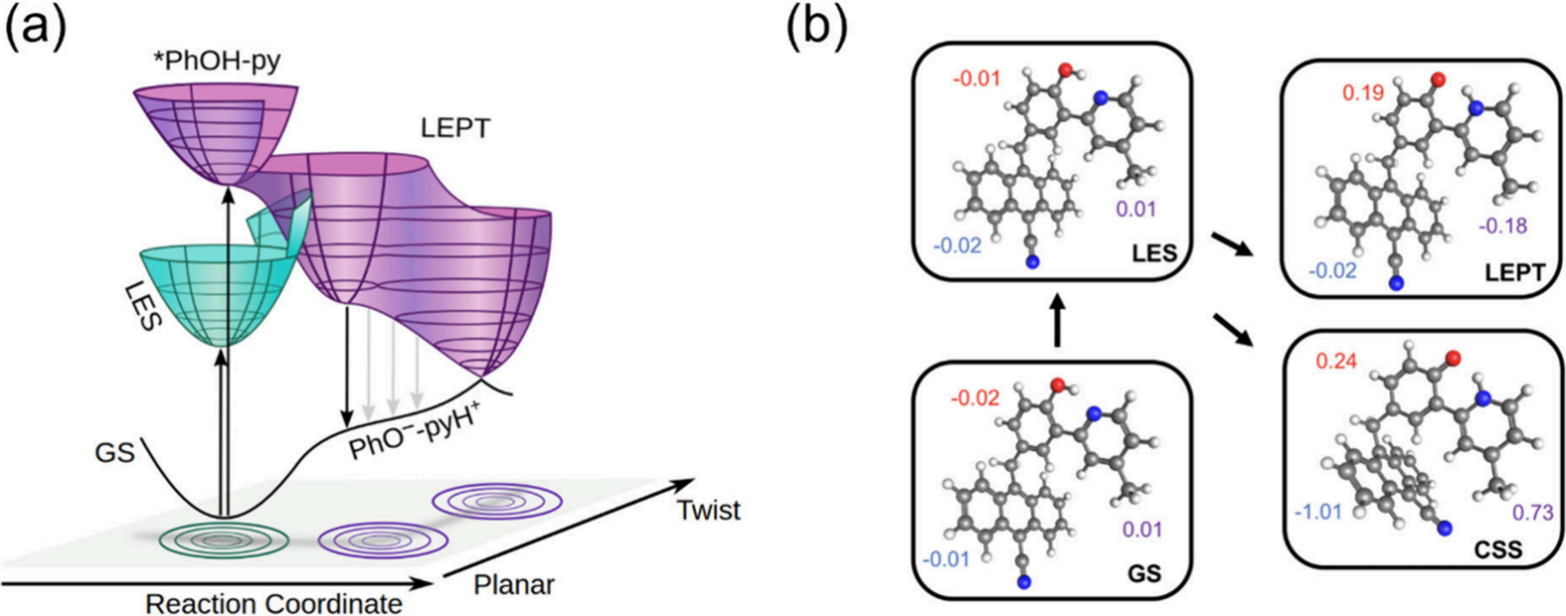
Potential energy surfaces and optimized structures for the relevant electronic states. (a) Schematic potential energy surfaces for the ESIPT and PCEnT reactions. ESIPT is barrierless on the excited-state potential energy surface (purple), whereas PCEnT from the LES to the LEPT state requires reorganization of the heavy nuclei to reach the intersection between the green and purple surfaces. (b) The optimized structures and natural bond orbital charges on the anthracene (blue), phenol (red), and pyridine (purple) fragments, excluding the charges from the methylene spacer. Reprinted from ref 14 under the CC BY license.

One way to describe PCEnT in the triads is that photoexcitation to the LES state is followed by thermal fluctuations of the solute and/or solvent that lead to resonance between the LES and LEPT state, allowing concerted EnT and PT (Figure 7). This description is analogous to PCET, which involves resonance between the LES and CSS, allowing concerted ET and PT. In contrast to PCET, however, no theoretical framework for analyzing the mechanisms and rate constants of PCEnT existed when it was discovered in the context of the triads.

## Theory for Proton-Coupled Energy Transfer

To further understand this PCEnT mechanism, a general theory^39^ for PCEnT was developed, analogous to the previous theory for PCET^1,11,17^ with inspiration from Förster/Dexter theory^40,41^ for conventional EnT. In this case, the vibronically nonadiabatic PCEnT rate constant has the form

3 $$k_{\mathrm{PCEnT}}\propto \underset{\mu }{\sum }\underset{\nu }{\sum }P_{\mu }V_{\mu \nu }^{2}I_{\mu \nu }$$

where *I*_μν_ is the spectral convolution integral. For the triad, PCEnT corresponds to the transition from the LES to the LEPT state, and the summations over μ and υ correspond to proton vibrational states on the LES and LEPT state, respectively. In the simplest case, which was shown to apply to the triad, *I*_μν_ becomes the spectral overlap between the donor emission and the acceptor absorption line shapes. Here the donor emission is from proton vibrational state μ on the LES to the lowest-energy proton vibrational state on the electronic ground state (GS) of the triad, and the acceptor absorption is from the lowest-energy proton vibrational state on the GS to proton vibrational state ν of the LEPT state.

As for PCET, the vibronic coupling is often of the form *V*_μv_ = *V* ^el^ *S*_μv_, which depends on the electronic coupling and the overlap integral between the reactant and product proton vibrational wave functions, corresponding to the LES and LEPT state, respectively. The electronic coupling for EnT can be estimated using Förster or Dexter theory in certain cases. Similar to PCET theory, the contribution from each pair of reactant/product vibronic states (μ,ν) depends on the balance among three quantities, but in this case the third quantity is the spectral overlap integral. Even when there is no observable spectral overlap between the donor emission and acceptor absorption spectra, PCEnT can occur if it is dominated by vibronic states that do not contribute significantly to the emission and/or absorption spectra.

Application of this theory to triad **1** ^42^ explained how PCEnT can occur even when there is no observable spectral overlap between the donor (An*) emission and acceptor (PhOH-py) absorption, corresponding to the LES emission and LEPT absorption, respectively (Figures 5 and 8). In this case, the (0,0) pair of vibronic states dominates PCEnT from the LES to the LEPT state, even though the overlap *S*_μυ_ between the proton vibrational wave functions is very small, because the spectral overlap *I*_μυ_ significantly favors the (0,0) pair (Figure 8). From a physical perspective, EnT requires PT to lower the energy of the LEPT state for sufficient spectral overlap. However, the υ = 0 LEPT vibronic state contributes negligibly to the acceptor absorption spectrum (i.e., absorption from the GS to the LEPT state) due to the small Franck–Condon overlap associated with proton transfer. Thus, PCEnT is dominated by vibronic states that do not contribute significantly to the LEPT absorption spectrum.

**Figure 8:**
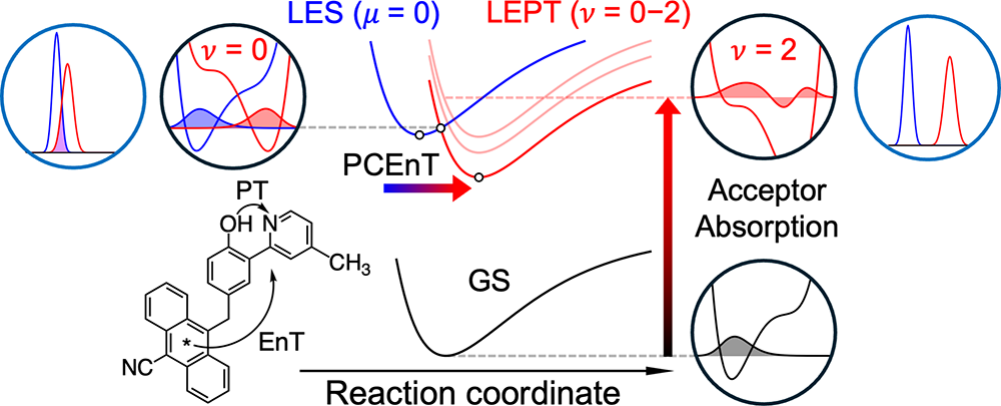
Theoretical description of PCEnT in triad **1**. The electron–proton vibronic potential energy surfaces for the GS (black), LES (blue), and LEPT state (red) as functions of the reaction coordinate, typically dominated by solute structural changes (center). Each stacked curve for the LEPT state corresponds to a different proton vibrational state, but only the curve corresponding to the lowest-energy proton vibrational state is shown for the LES and GS. The proton potentials and vibrational wave functions corresponding to the dominant reactant/product vibronic state pair for PCEnT are shown in the black circle at the upper left. This (0,0) pair dominates PCEnT despite the small proton vibrational wave function overlap integral because the spectral overlap integral significantly favors it. The line shape functions corresponding to the donor emission and acceptor absorption for PCEnT from the LES to the LEPT state are shown in the cyan circles [(0,0) upper left, (0,2) upper right], illustrating the greater spectral overlap for the (0,0) pair. The proton potentials and vibrational wave functions corresponding to the dominant pair of vibronic states for LEPT absorption are shown in the black circles on the right. The ν = 2 proton vibrational state dominates LEPT absorption because of the substantial Franck–Condon overlap. As the ν = 0 proton vibrational state does not contribute significantly to LEPT absorption, PCEnT can occur even with no apparent spectral overlap. Adapted from ref 42. Copyright American Chemical Society 2025.

Current work is directed toward elucidating the impact of solute motions on the PCET and PCEnT mechanisms in these triads.

## PCEnT in Other Systems

We recently showed that PCEnT can occur also in bimolecular reactions, and with different donor and acceptor units than those used in the triads described above.^43^ We used the well-known ESIPT compound 3HF (Figure 9) as the acceptor, for which the excited tautomer state is fluorescent also in fluid solution. The benzo­(ghi)­perylene (BPe) donor has a relatively long-lived singlet excited state, allowing for bimolecular encounter in room-temperature solution, and its lowest excitation energy is 0.25 eV below the excitation energy of the 3HF. PCEnT is indicated for the *BPe quenching by 3HF because the 3HF ground state absorption was much higher in energy than the donor excited state. Thus, PCEnT is not unique to the original triads but can occur more broadly. Moreover, a short covalent bond, as in the triads, is not required to mediate vibronic coupling between the donor and acceptor.

**Figure 9:**
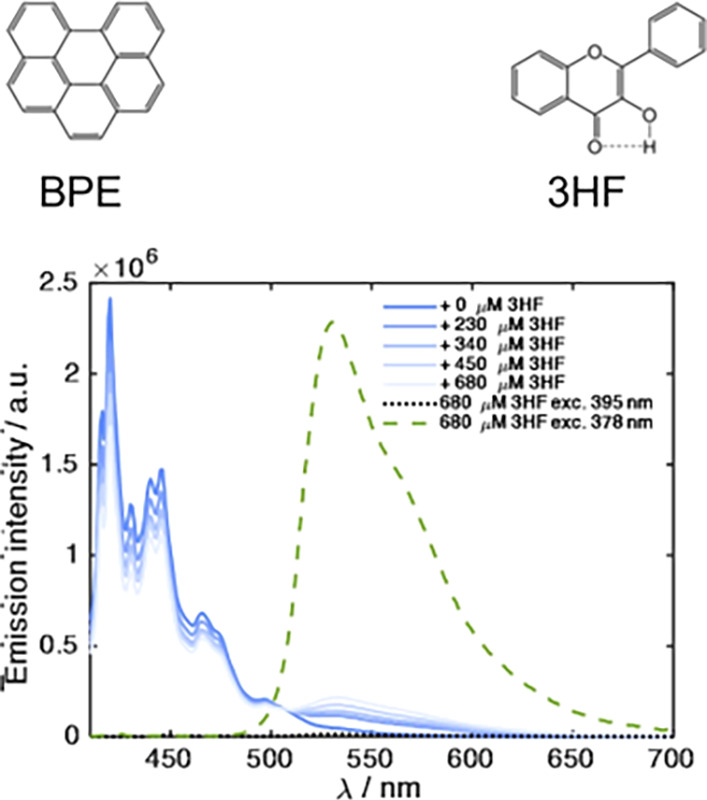
Structure of benzo­(ghi)­perylene (left) and 3HF (right), and their fluorescence spectra in decane. The 3HF fluorescence (520–650 nm) is sensitized by excitation of benzo­(ghi)­perylene at 390 nm. Also shown is the 3HF fluorescence upon direct excitation (green dashed) and when excited at 390 nm in the absence of the donor (black dotted); the latter is close to the baseline. Adapted from ref 43 under the CC BY license.

In the more polar solvents, quenching of *BPe by PCET was found to compete with PCEnT because the resulting CSS (BPe^–^ + 3HF^+^, detected by transient absorption) is more stable in those solvents. A similar competition was found in triads **1–2** in toluene at 298–140 K,^44^ where their CSS energies should lie between the values in polar solvent and in 77 K glass, as illustrated in Figure 6. While triad **2** only showed PCET CS and CR at all temperatures investigated, only about one-third of the excitations in **1** led to the CSS while the rest were quenched in a different reaction, which we proposed was PCEnT.

Overall, our results point to some of the challenges in observing PCEnT.^43^ First, for PCEnT to be favored over uncoupled energy transfer (EnT), the donor energy must be positioned at higher energy than the acceptor *keto–enol but below the *enol–enol excitation energy. The latter energy can be determined from the acceptor spectra, but the former corresponds to a nonvertical transition and requires calculations, and literature values can lack sufficient accuracy. Second, PCET can outcompete PCEnT for many donor–acceptor pairs. Third, if the *keto form is short-lived and not fluorescent, the PCEnT product will not be easily detected.

## Concluding Remarks

The anthracene-phenol-pyridine triads have a rich photophysical landscape, including the first example of the Marcus inverted region for PCET and the discovery of proton-coupled *energy* transfer, PCEnT. In these and other systems, the PCET and PCEnT pathways are competitive with PT, ET, EnT, and thermal excited state interconversion. The interplay among these pathways depends on the energies of the various states and the quantum mechanical couplings between the electronic and vibronic states.

We believe that inverted PCET, as well as PCEnT reactions, will prove to be generally occurring phenomena, as supported by the recent demonstration of bimolecular PCEnT with a different donor–acceptor pair.^43^ PCEnT has also been suggested to occur in light-activated CO release from flavonol, although no direct evidence was available.^45^ We have suggested that PCEnT might occur in biological systems with chromophores coupled to protons, but this remains to be shown. PCEnT could be a new mechanism to sensitize optically dark states or could be the basis for molecular switches. Long-lived PCET excited states due to the inverted region could also be valuable in artificial photosynthetic systems, as the fuel-forming reactions to be catalyzed also involve protons and electrons. We are excited to see how this rich field develops.
